# Clinical characterization and *in silico* structural analysis of microtubule-related gene variants in eleven patients

**DOI:** 10.3389/fgene.2026.1939857

**Published:** 2026-10-27

**Authors:** Yue Niu, Zhao Xu, Zongpu Zhou, Xianru Jiao, Zhixian Yang

**Affiliations:** Department of Pediatrics, Peking University People’s Hospital, Beijing, China

**Keywords:** clinical heterogeneity, epilepsy, microtubules, molecular mechanism, neurodevelopmental abnormalities

## Abstract

**Objective:**

This study aimed to analyze associations of Microtubules (MTs) mutations with nervous system phenotypes, summarize mutation characteristics of genes including *TUBA1A*, *TUBB2A*, *TUBB4A*, and *TUBGCP6*, explore clinical phenotypic diversity and molecular mechanisms, and expand the MTs genotype-phenotype spectrum.

**Methods:**

Eleven patients with MTs gene mutations were enrolled, and their clinical data were collected. Protein structure prediction was performed using AlphaFold3, and FoldX was employed to calculate mutation-induced energy changes, thereby evaluating protein stability and conformational impacts.

**Results:**

While global developmental delay was present in all individuals (11/11, 100%), epileptic seizures and structural brain abnormalities were variable phenotypic features, observed in 81.8% (9/11; including 8 with unprovoked epilepsy and 1 with an isolated febrile seizure) and 63.6% (7/11) of patients, respectively, with normal cranial MRI findings documented in four patients (36.4%). Four patients with *TUBA1A* were associated with severe cortical developmental malformations (lissencephaly, pachygyria), with two exhibiting dynamic developmental regression; four patients with *TUBB2A* consistently presented with severe developmental impairment and intractable epilepsy (including infantile epileptic spasm syndrome and Lennox-Gastaut syndrome), some displaying sound-induced reflex seizures, while none manifested developmental regression during the follow-up period. Two patients with *TUBB4A* showed marked differences in age of onset, and one presented with an isolated co-occurrence of systemic lupus erythematosus; one patient with *TUBGCP6* was mainly characterized by microcephaly and short stature without epilepsy. Across the 11 patients, a total of 12 variants were identified, comprising 10 missense variants, one non-canonical intronic/splice variant, and one copy number duplication. Prediction of protein structure and energy changes for the 10 missense variants showed varying degrees of increased free energy. Based on *in silico* structural predictions, we hypothesize that these mutations may potentially impair microtubule function by altering hydrogen bonds and conformation, with representative examples including *TUBA1A* Y172D (5.15 kcal/mol energy increase) and *TUBB4A* G10S (introduction of new hydrogen bond interactions).

**Conclusion:**

This study delineates the diverse clinical spectrum across patients harboring MTs variants: *TUBA1A* variants were predominantly observed in individuals with cortical malformations and potential developmental regression, *TUBB2A* with severe static neurodevelopmental delay and drug-resistant epilepsy, *TUBB4A* with motor impairment (with systemic lupus erythematosus noted in one individual as an unusual co-observation), and *TUBGCP6* with primary growth and developmental delay. In silico structural modeling (AlphaFold3 and FoldX) indicates that missense substitutions may perturb protein stability and local conformation (with ΔΔG values varying across sites); however, because these variants are predominantly identified in single individuals, these computational insights represent theoretical biophysical hypotheses rather than validated variant-specific phenotypic signatures. Further functional assays and larger cohorts are required to establish definitive genotype-phenotype correlations.

## 1 Introduction

Microtubules (MTs) are one of the four non-covalent polymers involved in several cellular functions, such as intracellular trafficking, cell division and motility (Maillard et al., 2023). The basic building block of MTs is the αβ-tubulin heterodimer. It assembles in a head-to-tail manner to form a polar protofilament, with α-tubulin exposed at one end and β-tubulin at the other end (Nogales, 2000). MTs are closely related to the occurrence and development of a variety of diseases, including tumors, nervous system diseases, and cardiovascular diseases.

Compared with other cell types, neurons rely more heavily on the proper functioning of microtubules to carry out their activities and form complex morphological structures (Mora-Bermúdez and Huttner, 2015). Mutations in MTs can lead to a variety of brain malformations, such as abnormal neuronal migration and organization, abnormal differentiation, and abnormal axon guidance (Cinarová and Tuszynski, 2019). Up to now, patients with variations in *TUBA1A*, *TUBA4A, TUBB2B*, *TUBB3*, *TUBB2A*, *TUBB*, and *TUBB4A* have been reported (Bahi-Buisson et al., 2014; Romaniello et al., 2014; Chang, 2015). Moreover, such mutations have also been found in the gamma (γ)-tubulin gene *TUBG1,* which can lead to diseases such as cortical dysplasia, epilepsy, and neurodevelopmental delay and regression (Ivanova et al., 2019).

Clinically, individuals with MTs mutations exhibit a series of phenotypes, ranging from severe intellectual disabilities and intractable epileptic seizures to learning difficulties and the absence of epileptic seizure (Oegema et al., 2015). However, current literature on MTs mainly focuses on reports of single genes, with neurological phenotypes primarily concentrated on cortical dysplasia (Poirier et al., 2010). In this article, we described multiple MTs genes to summarize various neurological phenotypes, including malformations of cortical development (MCD).

## 2 Ethics statement and methods

This study was a single-center, retrospective cohort study conducted at the Department of Pediatrics, Peking University People’s Hospital. The study protocol was reviewed and approved by the Biomedical Research Ethics Committee of Peking University People’s Hospital (Approval No. 2023PHB245-001). Written informed consent was obtained from the parents or legal guardians of all pediatric participants prior to inclusion in the study. All procedures adhered to the ethical standards of the Declaration of Helsinki. Between January 2018 and December 2024, pediatric patients evaluated for unexplained global developmental delay (GDD), intellectual disability, intractable epilepsy, or brain structural malformations were retrospectively screened. The inclusion criteria were as follows: (1) clinical presentation of neurodevelopmental abnormalities (including developmental delay, epileptic seizures, or malformations of cortical development); (2) identification of rare single-nucleotide variants (SNVs), indels, or copy number variations (CNVs) involving microtubule-related genes (*TUBA1A*, *TUBB2A*, *TUBB4A*, or *TUBGCP6*) confirmed by trio-based whole-exome sequencing (WES) and validated by Sanger sequencing or quantitative methods; (3) availability of complete clinical history, developmental milestone records, and follow-up neurological evaluations. The exclusion criteria included: (1) patients with secondary acquired brain injuries (e.g., severe neonatal hypoxic-ischemic encephalopathy, documented central nervous system trauma, or intrauterine infection); (2) patients carrying other co-existing primary pathogenic variants known to cause monogenic neurodevelopmental syndromes; (3) patients lost to follow-up or with incomplete neuroimaging/clinical records. A total of 11 eligible probands fulfilling these criteria were consecutively retrieved and enrolled for detailed genotype-phenotype and structural analyses. To evaluate neonatal anthropometric status, birth weight, length, and head circumference were converted into gestational-age- and sex-adjusted percentiles and z-scores using the Chinese neonatal intrauterine growth curves. Measurements between the 10th and 90th percentiles (or within ±2 SD) were considered within the normal range.

A total of 11 SNVs/small alterations (consisting of 10 missense variants and one non-canonical intronic alteration) and one large CNV duplication were characterized across the 11 probands (totaling 12 variants; Table 1). All variants were annotated against canonical NCBI RefSeq transcripts (*TUBA1A*: NM_006009.4; *TUBB2A*: NM_001069.3; *TUBB4A*: NM_006087.4; and *TUBGCP6*: NM_020461.4). Variant pathogenicity was systematically evaluated in accordance with the ACMG/AMP and ClinGen technical standards. To systematically determine the novelty of identified variants, population and disease-specific variant repositories—including the Genome Aggregation Database (gnomAD v4.1.0/v2.1.1), ClinVar, the Human Gene Mutation Database (HGMD Professional 2024.4), Mastermind Genomic Search Engine, and DECIPHER—were queried. In addition, an extensive literature search was conducted across PubMed, Web of Science, and Google Scholar using gene symbols combined with nucleotide/protein alterations or codon positions. Variants were classified as 'novel’ or 'previously unreported’ if they were entirely absent from clinical disease databases (ClinVar and HGMD) and published peer-reviewed medical literature. The most recent comprehensive search and variant assessment was performed on 31 December 2024. Nine missense variants identified in *TUBA1A*, *TUBB2A*, and *TUBB4A* were classified as pathogenic or likely pathogenic. All were *de novo* (including two post-zygotic mosaic cases: P2 and P6) and absent from the gnomAD population database. In P10, chromosomal microarray/WES-CNV analysis revealed a *de novo* 2.36 Mb pathogenic contiguous gene duplication on chromosome 19p13.3–p13.2 (genomic build GRCh38/hg38, chr19:4816250–7174,742) encompassing TUBB4A along with multiple adjacent genes. This CNV was classified as pathogenic in accordance with ClinGen CNV classification guidelines (criteria: 1A + 2A + 3B + 4H). In P11, compound heterozygous candidate variants in *TUBGCP6* were identified (maternal c.5050A>G, p. Ile1684Val; paternal c.1492-35G>A). Given the absence of experimental transcript validation, the non-canonical intronic alteration was conservatively designated as a variant of uncertain significance (VUS), and the inheritance was framed in accordance with autosomal recessive microcephaly spectrum disorders. Full details of ACMG criteria attribution, population allele frequencies, and *in silico* pathogenicity scores are summarized in Table 1.

**TABLE 1:** Detailed genetic profile and ACMG/AMP pathogenicity classification of identified variants.

Patient	Gene	Transcript ID	Nucleotide change (cDNA)	Amino acid change (protein)	Inheritance	gnomAD freq (v4.1)	In silico (revel/cadd)	ACMG criteria applied	ACMG classification
P1	*TUB A1A*	NM_006009.4	c.524C>T	p.Pro175Leu	*De novo*	Absent	0.812/28.4	PS2 + PM1 + PM2_Supp + PP3	Pathogenic
P2	*TUBA1A*	NM_006009.4	c.1131G>A	p.Met377Ile	Mosaic	Absent	0.745/25.8	PM1 + PM2_Supp + PM6 + PP3	Likely pathogenic
P3	*TUBA1A*	NM_006009.4	c.449C>T	p.Thr150Ile	*De novo*	Absent	0.892/31.0	PS2 + PM1 + PM2_Supp + PP3	Pathogenic
P4	*TUBA1A*	NM_006009.4	c.514T>G	p.Tyr172Asp	*De novo*	Absent	0.934/32.0	PS2 + PM1 + PM2_Supp + PP3	Pathogenic
P5	*TUBB2A*	NM_001069.3	c.743C>T	p.Ala248Val	*De novo*	Absent	0.784/27.2	PS1 + PS2 + PM1 + PM2_Supp + PP3	Pathogenic
P6	*TUBB2A*	NM_001069.3	c.609T>G	p.Asp203GLu	Mosaic	Absent	0.826/29.1	PS2 + PS2 + PM1 + PM2_Supp + PP3	Likely pathogenic
P7	*TUBB2A*	NM_001069.3	c.758C>A	p.Arg262His	*De novo*	Absent	0.915/32.0	PS2 + PM1 + PM2_Supp + PP3	Pathogenic
P8	*TUBB2A*	NM_001069.3	c.758C>A	p.Arg391His	*De novo*	Absent	0.867/28.9	PS2 + PM1 + PM2_Supp + PP3	Pathogenic
P9	*TUBB4A*	NM_006087.4	c.28G>A	p.Gly10Ser	*De novo*	Absent	0.791/26.5	PS2 + PM1 + PM2_Supp + PP3	Pathogenic
P10	*TUBB4A*	NM_006087.4	chr19:4816250–7174,742 duplication (2.36 Mb)	-	*De novo*	NA	NA	1A + 2A + 3B + 4H (ClinGen CNV guidelines)	Pathogenic (CNV)
P11	*TUBGCP6*	NM_020461.4	c.5050A>G	p.Ile1684Val	Maternal	0.00003	0.518/22.1	PM1 + PM2_Supp + PP3	VUS/Likely Patho (provisional)
P11	*TUBGCP6*	NM_020461.4	c.1492-35G>A	Intronic/Splicing	Paternal	0.0012	<0.05	BP4 + BP7 + BS1_Supp	VUS (provisional)

Abbreviations: RefSeq, NCBI, reference sequence; gnomAD, genome aggregation database; ACMG, american college of medical genetics and genomics; CNV, copy number variation; VUS, variant of uncertain significance; NA, not applicable. Novelty assessment was conducted against gnomAD (v4.1.0), ClinVar, HGMD, Professional (release 2024.4), and PubMed up to 31 December 2024.

Protein primary sequences and standard isoform annotations were retrieved from the UniProt database: TUBA1A (UniProt ID: Q71U36, Isoform 1), TUBB2A (UniProt ID: Q13885, Isoform 1), TUBB4A (UniProt ID: P68371, Isoform 1), and TUBGCP6 (UniProt ID: Q96RG6, Isoform 1). Protein 3D structures were predicted using the AlphaFold3 web server (version released in 2024/2025) in monomer prediction mode under default parameters (Roy and Al-Hashimi, 2024). For each protein, five models were generated, and the top-ranking model (rank_1) with the highest overall confidence score (average pLDDT > 90 across structured domains) was selected as the template for downstream analysis. To evaluate the thermodynamic impact of missense variants, FoldX (version 5.0) was employed (Sapozhnikov et al., 2023). Prior to mutagenesis, the wild-type PDB structures derived from AlphaFold3 were subjected to energy minimization and side-chain optimization using the FoldX Repair PDB command to eliminate steric clashes. The BuildModel function was subsequently executed to generate mutant structures and calculate the free energy change (ΔΔG). Each mutation was modeled across 5 independent BuildModel runs, and the average ΔΔG value was reported. Here, ΔΔG is explicitly defined as the predicted change in protein folding thermodynamic stability (ΔGmutant−ΔGwild-type) in units of kcal/mol. A positive ΔΔG value (ΔΔG>0) represents a predicted decrease in folding stability (destabilization), whereas a negative value indicates increased stability.

## 3 Results

A total of 11 patients harboring variants in MTs-related genes were included, comprising four with *TUBA1A*, four with *TUBB2A*, two with *TUBB4A*, and one with a *TUBGCP6* alteration. All 11 patients were born at term (or appropriate preterm) with birth weight, length, and head circumference all falling within the normal range (between the 10th and 90th percentiles, adjusted for gestational age and sex based on standard Chinese neonatal growth references). Across the cohort, global developmental delay (GDD) was universally present (11/11, 100%). Conversely, epileptic seizures and structural neuroimaging abnormalities exhibited marked clinical heterogeneity: seizures occurred in 9 of 11 patients (81.8%; 8 with active epilepsy syndromes and 1 with an isolated febrile seizure), while neuroimaging abnormalities were identified in 7 of 11 patients (63.6%), with cranial MRI demonstrating normal findings in the remaining four individuals (36.4%). The comprehensive individual clinical profiles are summarized in Table 2.

**TABLE 2:** Clinical characteristics of the Microtubules gene mutations.

Patient	Gene	Sex	Nucleotide sequence variation	Protein sequence variation	Inheritance	Age of onset	Epilepsy syndrome	Seizure type	ASMs	EEG	Brain imaging (age)	Other features
P1	TUBA1A	M	c.524C>T	P175L	*De novo*	6months	No	-	None	Normal (6years)	Mild leukomalacia (3years)	GDD
P2	TUBA1A	F	c.1131G>A	M377I	Mosaic	3months	No	Focal seizure	VPA, lev, OXC	Multifocal discharge (7y8m)	Cerebellar hypoplasia (6years)	GDD
P3	TUBA1A	F	c.449C>T	T150I	*De novo*	4months	No	Focal seizure	TPM, OXC	Background Deterioration (1year) Bilateral posterior discharges (2years)	Lissencephaly (2m)	GDD, microcephaly, micrognathia, hypertonia
P4	TUBA1A	F	c.514T>G	Y172D	*De novo*	6months	LGS	Focal seizure/tonic seizure	Lev, LTG, VPA	Left posterior discharges (1y3m)	Bilateral frontal parietal lobe giant gyrus malformation (3m)	GDD, congenital vertical talus
P5	TUBB2A	M	c.743C>T	A248V	*De novo*	4months	No	Febrile seizure	None	Normal (1y7m)	Normal	GDD, stereotyped action
P6	TUBB2A	F	c.609T>G	D203E	Mosaic	20days	LGS	Focal seizure/tonic seizure (voice evoked)	VPA, lev, LTG	Generalized discharges (2y4m)	Deepening of the fronto-temporo-parietal sulcus, brain atrophy	GDD
P7	TUBB2A	F	c.758C>A	R262H	*De novo*	3days	IESS	Spasm seizure/myoclonic seizure	ACTH, VPA, LCM, TPM	Hypsarrhythmia (1y2m)	Multiple cerebellar gyrus malformation	GDD
P8	TUBB2A	F	c.1172C>A	R391H	*De novo*	8months	IESS	Spasm seizure (startle evoked)	ACTH, VPA, lev, VGB, VGB, TPM	Hypsarrhythmia (8m)	Normal	GDD
P9	TUBB4A	F	c.28G>A	G10S	*De novo*	11year	NA	Focal seizure	VPA, CLB	Rolandic discharges (21yearsear)	Normal	Lagging in intelligence and gross motor, ASD, lack of coordination, obvious motor regression, hypotonia and SLE
**P10**	TUBB4A	F	chr19:4816250–7174,742 duplicate (TUBB4A)	-	*De novo*	1year4months	NA	Focal seizure	LTG	Rolandic discharges70%(7years)	Reduced volume of the cerebral gyri and widened and deepened cerebral sulci in both cerebral hemispheres	GDD, open - mouthed facial appearance, hypotonia, right lower limb limp
**P11**	TUBGCP6	M	c.5050A>G, c.1492-35 (IVS6) G>A	I1684V	Compound heterozygosity	1year3months	NA	No	None	None	Normal	GDD, short stature and microcephaly

P: patient, NA: not available, IESS: infantile epileptic spasm syndrome; VPA: valproic acid; LTG: lamotrigine; OXC: oxcarbazepine; TPM: topiramate; LCM: lacosamide; ACTH: adrenocorticotropic hormone; VGB: vigabatrin; GDD: global developmental delay, ASD: autism spectrum disorder; SLE: systemic lupus erythematosus; IESS: infantile epileptic spasm syndrome; LGS: Lennox-Gastaut Syndrome; y: years; m; months.

### 3.1 Clinical features

#### 3.1.1 *TUBA1A* patients

Four patients (P1–P4; three females, one male) presented with early-onset GDD with onset between 3 and 6 months of age. While all patients exhibited prominent gross motor and cognitive deficits, developmental regression was a prominent feature in two individuals: P1 experienced motor deterioration following a febrile episode at 6 months, and P3 showed cognitive and motor regression after 2 years of age. Three of the four patients (P2–P4) developed drug-resistant focal seizures. P4 additionally experienced tonic seizures, fulfilling the diagnostic criteria for Lennox-Gastaut syndrome (LGS). Despite multi-drug anti-seizure medication (ASM) regimens, daily refractory seizures persisted in all three affected individuals. P1 remained completely seizure-free. MRI revealed a diverse spectrum of malformations: classical severe MCD was observed in P3 (lissencephaly) and P4 (bilateral frontoparietal pachygyria), whereas P2 exhibited cerebellar hypoplasia, and P1 showed only mild periventricular leukomalacia (Figure 1). Individual milestone timelines and associated systemic features (e.g., micrognathia in P3 and vertical talus in P4) are detailed in Table 2.

**FIGURE 1:**
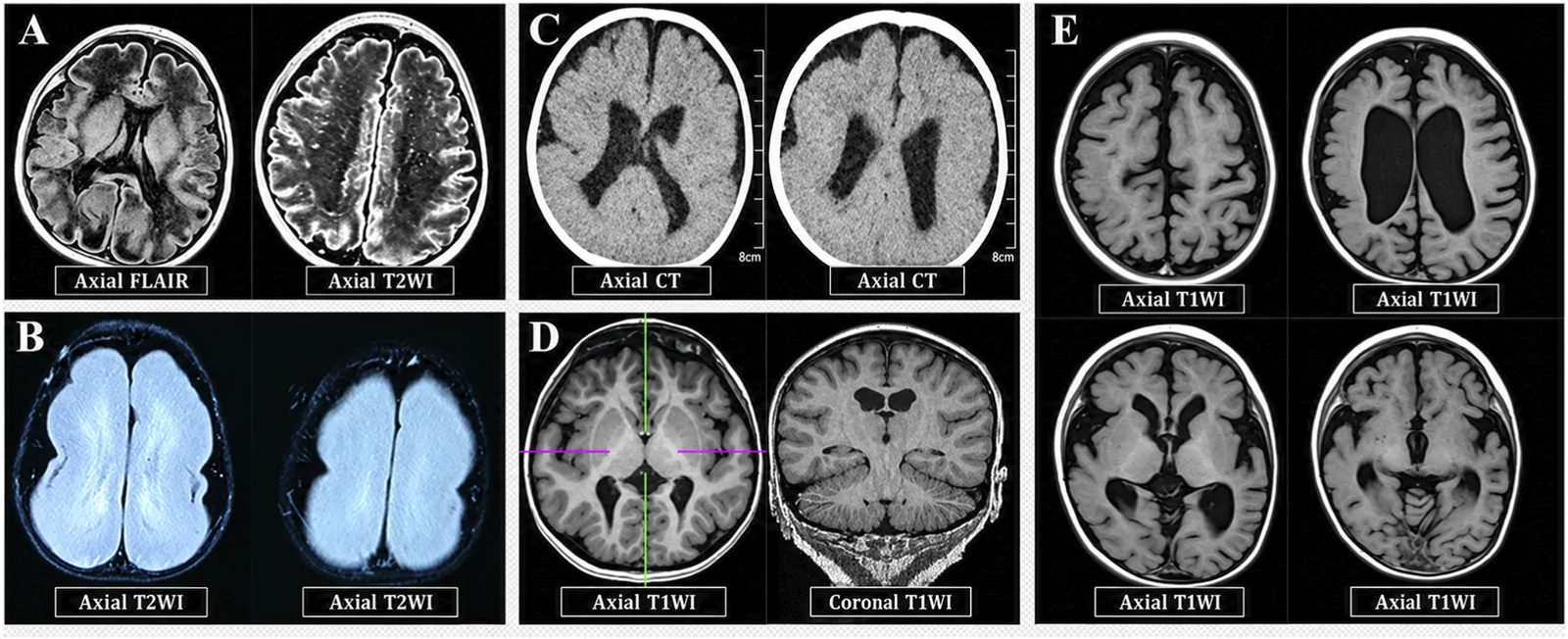
Neuroimaging spectrum of patients 1, 3, 4, 6, and 7. **(A)** P1: Periventricular leukomalacia. **(B)** P3: Agyria. **(C)** P4: Frontoparietal gyral malformations. **(D)** P7: Polymicrogyria. **(E)** P6: Deepening of the fronto-temporo-parietal sulci and cerebral atrophy.

#### 3.1.2 *TUBB2A* patients

Four patients (P5–P8; three females, one male) presented with early developmental impairment, with disease onset ranging from the neonatal period (day 3) to 4 months of age. Severe delay across gross motor, expressive language, and social domains was evident in all cases; independent ambulation and command comprehension were significantly impaired or delayed. None exhibited dynamic regression during follow-up. All four patients experienced seizures. Three individuals (P6–P8) developed early-onset. P6 presented with refractory focal and tonic seizures manifesting as LGS, while P7 and P8 presented with infantile epileptic spasm syndrome (IESS) accompanied by hypsarrhythmia, both relapsing around 3 years of age into drug-resistant epilepsy (Figure 2). Notably, acoustic/voice-induced reflex seizures were documented in both P6 and P8. Structural brain alterations were present in two of four probands: P6 displayed deepened fronto-temporo-parietal sulci with progressive cerebral atrophy (Figure 1), and P7 showed multiple cerebellar gyral malformations. Brain MRI was normal in P5 and P8.

**FIGURE 2:**
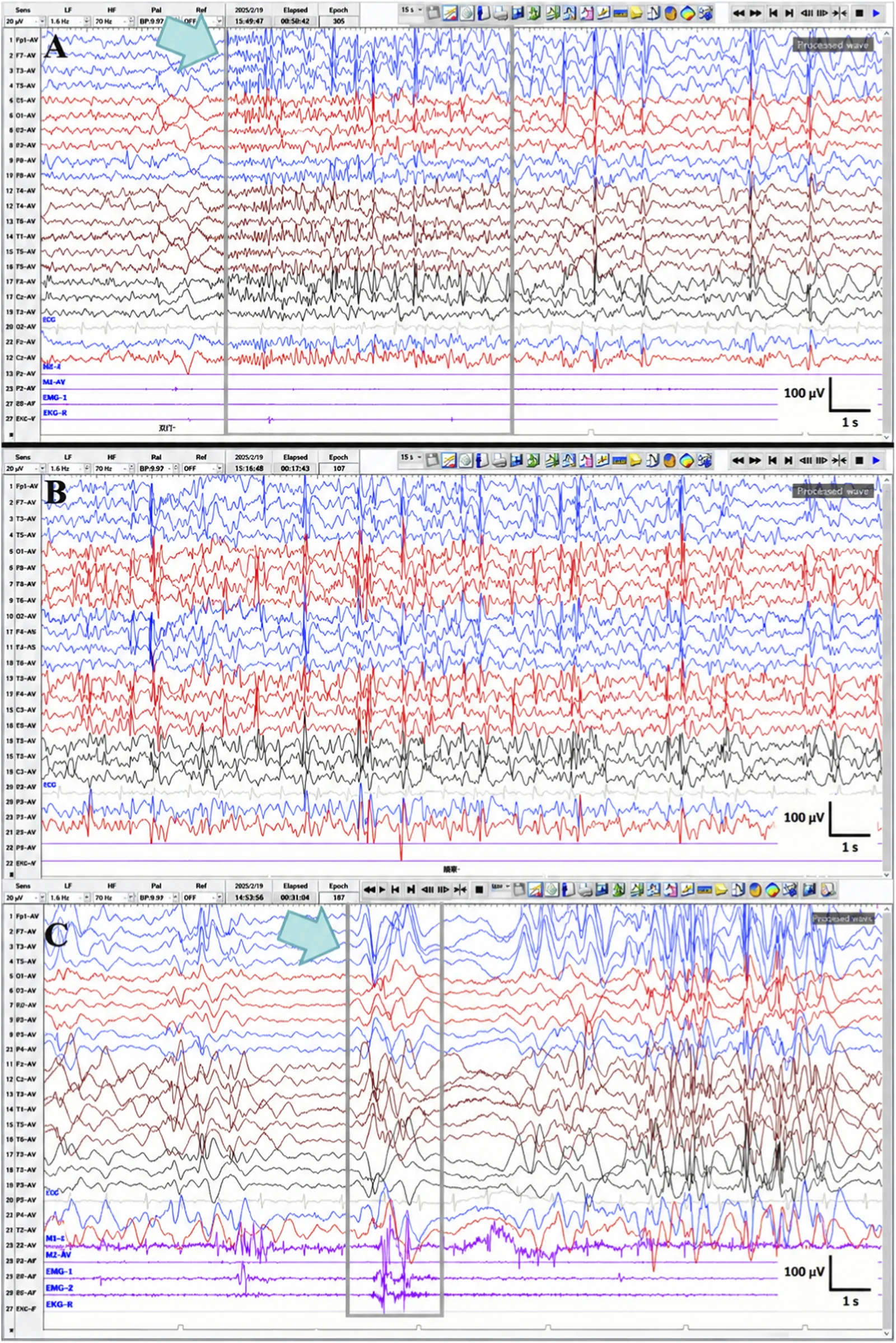
**(A)** Patient P4 (LGS) in the awake state, showing a tonic seizure with generalized paroxysmal fast activity (GPFA; arrow indicates seizure onset, grey box outlines ictal discharges).**(B)** Interictal generalized slow spike-and-wave discharges (<2.5 Hz) in Patient P4 during wakefulness.**(C)** Patient P7 in a drowsy state, exhibiting epileptic spasms (arrow and grey box indicate typical discharges). EEG traces were recorded using a standard 10–20 average reference montage (filter settings: LF 1.6 Hz, HF 70 Hz). Scale bars in the bottom right corner represent amplitude (100 μV) and time (1 s). The framed boxes indicate annotated seizures in the pediatric patients.

#### 3.1.3 *TUBB4A* patients

Two patients carry *TUBB4A* mutations. The age of onset differed significantly between the two cases: P9 had an onset at 11 years old, while P10 had an onset at 1 year and 4 months old. P9 presented only with mild intellectual disability and autism spectrum disorder. At the last follow-up at 21 years old, the patient walked with a slight limp and was thin. She was sensitive to sunlight, had anemia, and carried an independent clinical diagnosis of systemic lupus erythematosus. At the last follow-up at 6 years and 10 months old, P10 had a limp in the right lower limb when walking and easily fell down. The muscle strength of the right lower limb was grade III, and that of the left lower limb was grade V. The developmental quotient (DQ) was less than 50 points, indicating severe intellectual developmental delay.

Both patients presented with focal seizures. For P9, seizures were controlled with oral lamotrigine for 6 years; however, at the last follow-up at 21 years of age, frequent focal seizures originating from the centroparietal recurred. P10 experienced only two focal seizures, which were controlled following oral administration of valproate acid and clobazam. During 3 years of follow-up, the child remained seizure-free. Notably, both patients exhibited similar EEG findings, characterized by Rolandic discharges predominantly in the centroparietal regions, with P10 showing the highest discharge rate of up to 70% at 8 years old.

#### 3.1.4 *TUBGCP6* patients

P11 with *TUBGCP6* was able to walk at 1 year and 3 months old and could say “mom” and “dad” at 2 years old. Currently, at 2 years and 6 months old, he has a height of 88 cm (<-2SD), weight of 11 kg (<-2SD), and head circumference of 42 cm (-3SD). He still cannot speak simple sentences and only uses simple reduplicated words. No epileptic seizures have occurred to date, and no abnormalities have been observed in other systems such as the digestive and ocular systems.

### 3.2 Mutation and protein structural change

Variant pathogenicity was classified according to ACMG/AMP and ClinGen technical guidelines (Table 1). The implicated tubulin genes (*TUBA1A*, *TUBB2A*, and *TUBB4A*) follow an autosomal dominant inheritance pattern, whereas *TUBGCP6* exhibits autosomal recessive inheritance. Across the 11 probands, 12 variants were characterized: 10 missense variants, one non-canonical intronic alteration, and one chromosomal microduplication. Specifically, nine patients (P1–P9) carried heterozygous *de novo* missense variants (including post-zygotic mosaicism confirmed in P2 and P6), P10 harbored a *de novo* 2.36 Mb contiguous duplication encompassing *TUBB4A*, and P11 carried compound heterozygous variants in *TUBGCP6* (maternally inherited c.5050A>G [p.Ile1684Val] and paternally inherited c.1492-35G>A). Pedigree analysis and parental segregation across all 11 families are illustrated in Figure 3. The genetic details and pathogenicity assessments are summarized in Table 1, 2, and *in silico* modeled structural impacts of the missense substitutions are illustrated in Figure 4.

**FIGURE 3:**
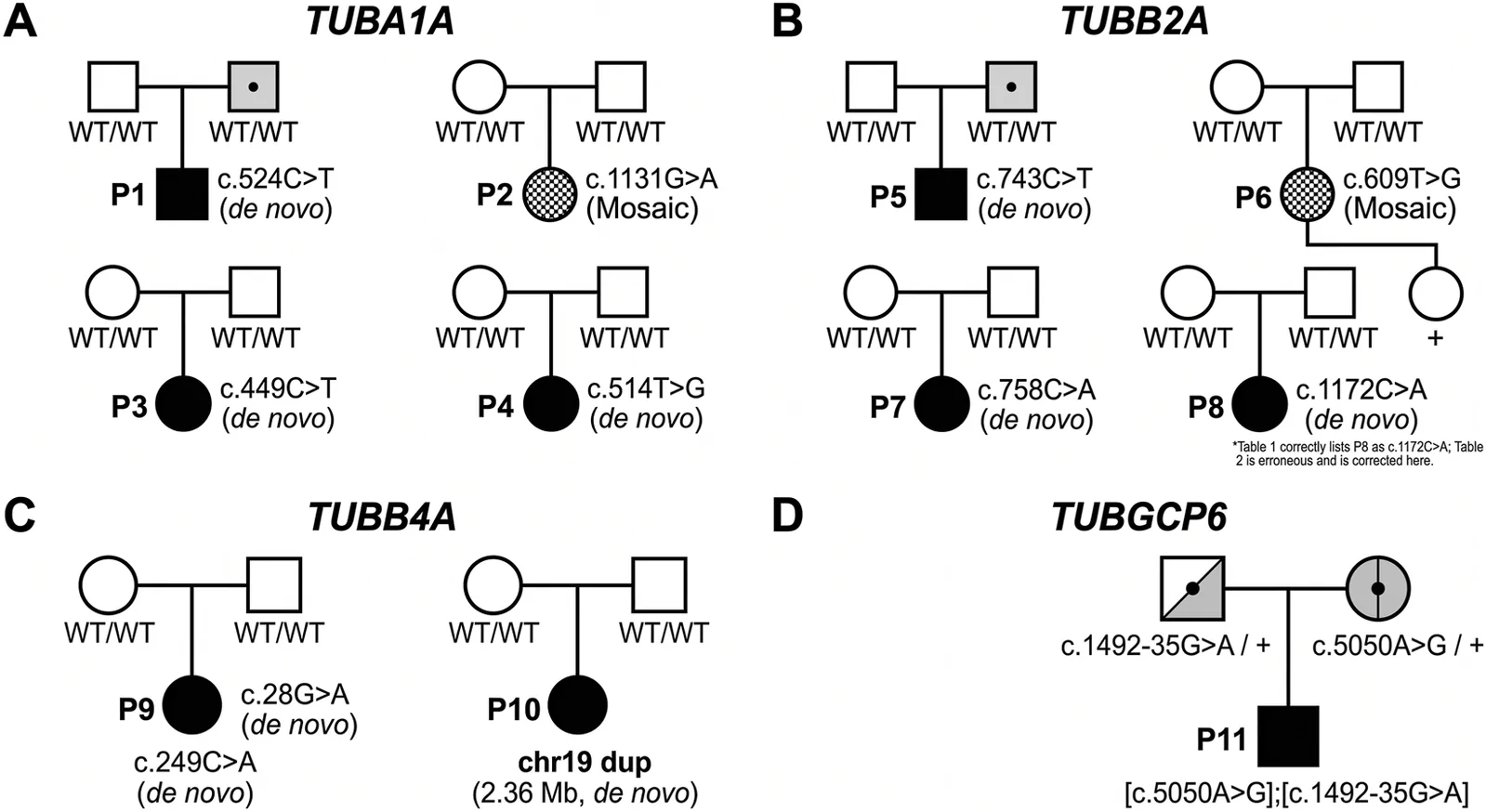
Pedigrees and variant segregation of the 11 families with microtubule-related gene variants (P1–P11). **(A)** Families with *TUBA1A* variants (P1–P4). **(B)** Families with *TUBB2A* variants (P5–P8). **(C)** Families with *TUBB4A* variants (P9–P10). **(D)** Family with compound heterozygous *TUBGCP6* variants (P11). Squares represent males and circles represent females; filled symbols indicate affected individuals, open symbols represent unaffected individuals, and split/hatched symbols represent post-zygotic mosaicism. Arrows denote probands. Genotypes are indicated below each tested individual (+: wild-type allele; mut: mutated allele; dup: duplication).

**FIGURE 4:**
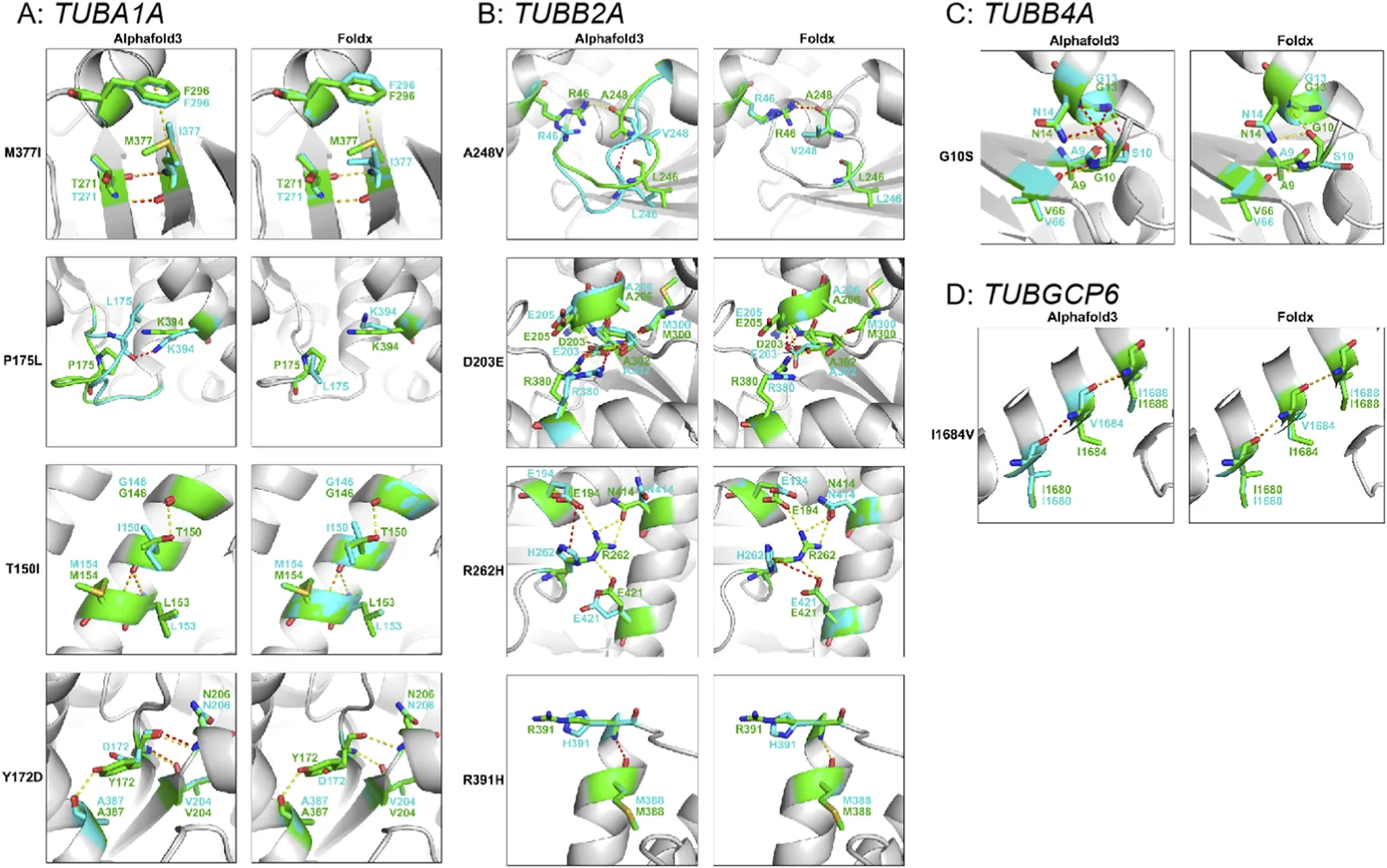
The spatial changes in protein structure at the missense locus of Microtubules mutation. **(A)** The missense mutation site alteration in TUBA1A. **(B)** The missense mutation site alteration in TUBB2A. **(C)** The missense mutation site alteration in TUBB4A. **(D)** The missense mutation site alteration in TUBGCP6.

#### 3.2.1 *TUBA1A* variant site protein structural change

TUBA1A harbors four missense variants with distinct stability and conformational impacts via FoldX energy calculations and structural modeling: M377I elevates energy by ΔΔG 1.44 kcal/mol, preserving T271 main-chain hydrogen bonds but losing F296 Pi-Sulfur interaction in both models; P175L increases energy by ΔΔG 1.04 kcal/mol, inducing loop changes in AlphaFold3 that form a novel L175-K394 hydrogen bond; T150I raises energy by ΔΔG 0.50 kcal/mol, maintaining M154/L153 main-chain bonds while losing G146 side-chain hydrogen bond; Y172D causes the largest energy increase (ΔΔG 5.15 kcal/mol), retaining V204/N206 main-chain bonds but reducing A387 side-chain hydrogen bonding. These mutations differentially modulate protein stability and intermolecular interactions via distinct structural pathways, exhibiting model-dependent conformational variations.

#### 3.2.2 *TUBB2A* variant site protein structural change

The four missense variants of *TUBB2A* exhibit distinct stability effects and conformational changes through FoldX energy calculations and structural modeling: The A248V mutation increases energy by ΔΔG 0.76 kcal/mol, with conformational changes in the loop region at position 248 in the AlphaFold3 model leading to the loss of the hydrogen bond between the A248 main-chain oxygen and R46 side-chain nitrogen, whereas this hydrogen bond is retained in the FoldX model; The D203E mutation increases energy by ΔΔG 4.38 kcal/mol, with stable main-chain hydrogen bonds but model-specific differences in side-chain interactions—in AlphaFold3, hydrogen bonds with R380 are reduced from two to one with loss of E205 interaction, while in FoldX, a second hydrogen bond with E205 is gained; The R262H mutation increases energy by ΔΔG 3.38 kcal/mol, with complete loss of side-chain hydrogen bonds with E194, N414, and E421; instead, H262 forms a pi-anion interaction with E194 in AlphaFold3 and with E421 in FoldX. The R391H mutation increases energy by ΔΔG 1.31 kcal/mol, with unchanged main-chain hydrogen bonds with M388; neither the original R391 nor mutant H391 side chains show significant interactions with neighboring residues. These results suggest that these *TUBB2A* missense variants may potentially alter microtubule-related properties by affecting protein stability and key site interactions, with conformational details varying across different modeling methods.

#### 3.2.3 *TUBB4A* variant site protein structural change

Patient P10 carries a *de novo* 2.36 Mb pathogenic microduplication at chromosome 19p13.3–p13.2 (genomic build GRCh38/hg38, chr19:4816250–7174,742) involving *TUBB4A* and adjacent genomic content. Due to the multi-gene nature of this region, the clinical presentation must be interpreted as a contiguous gene duplication rather than an isolated single-gene variant.” In the G10S, FoldX-BuildModel calculations indicate that the mutation increases the overall energy by ΔΔG 4.31062 kcal/mol. Structural analysis reveals that in the structural model of the wild-type protein, G10 forms three hydrogen bond interactions with the adjacent N14 and V66. In the mutant model generated by AlphaFold3, the mutated S10 retains these three hydrogen bonds but additionally forms two new hydrogen bonds with G13 and A9.

#### 3.2.4 *TUBGCP6* variant site protein structural change

Patient P11 carried compound heterozygous variants in *TUBGCP6:* a maternally inherited missense variant (c.5050A>G, p. Ile1684Val) and a paternal intronic variant situated near the splice acceptor region (c.1492-35G>A). In silico splicing evaluations via SpliceAI predicted negligible alterations to canonical splice motifs (delta scores ≤0.01). Because splice-region alterations do not directly alter the primary amino acid sequence in the absence of transcript-level validation, structural modeling was concentrated on the missense substitution p. Ile1684Val. FoldX calculations indicated a mild free-energy increase of ΔΔG 0.975 kcal/mol. In structural models generated by AlphaFold3 and FoldX, the main-chain hydrogen bonds between residue 1684 and neighboring residues (Ile1680 and Ile1688) remained intact relative to the wild-type protein, with no aberrant novel side-chain interactions introduced.

## 4 Discussion

This study analyzed the clinical and molecular characteristics of 11 patients with various MTs mutations, revealing an association between MTs gene variants and neurological phenotypes, with differences in clinical phenotypes observed among different genetic variants. This study summarizes the clinical and molecular characteristics of 11 patients with various microtubule-related gene variants. Rather than establishing a definitive, systematic genotype-phenotype matrix, this case series describes individual phenotypic manifestations associated with rare variants in *TUBA1A*, *TUBB2A*, *TUBB4A,* and *TUBGCP6*, while utilizing exploratory *in silico* tools to visualize potential structural impacts (Maillard et al., 2023; Hebebrand et al., 2019).

As a member of the α-tubulin family, TUBA1A is the gene with the most variant cases among MTs genes. It participates in microtubule assembly and stabilization during neuronal migration, regulates microtubule dynamics via GTP, influences neuronal polarity, axon growth, and synapse formation, and serves as a key molecule for cortical development and brain structure. (Tischfield and Engle, 2010). Previous studies have reported hundreds of patients with *TUBA1A* variants (Hebebrand et al., 2019; Ren et al., 2024). On this basis, the current study reports three previously unreported loci (P175L, M377I, Y172D), expanding the genotype-phenotype spectrum. Research shows that among 107 patients with *TUBA1A*, 98% of the children have global developmental delay, 76% have microgyria, 70% have lissencephaly-pachygyria malformation; 72.5% of the children have epilepsy, mainly focal seizures, which can present with LGS and sound-stimulated reflex seizures (Hebebrand et al., 2019). The four patients in this study also presented with relevant phenotypes, consistent with previous literature (Hebebrand et al., 2019; Schröter et al., 2022).

Notably, previous literature indicates that variants affecting the C-terminus or GTP-binding regions of *TUBA1A* (such as Arg402 and Ala174) are frequently associated with severe diffuse cortical malformations (e.g.,), lissencephaly, pachygyria), whereas variants in the N-terminus or non-critical interfaces tend to exhibit milder or more restricted structural abnormalities (Schröter et al., 2022). In our cohort, the four individuals harboring *TUBA1A* variants exhibited heterogeneous neuroimaging findings, ranging from lissencephaly (P3) and pachygyria (P4) to cerebellar hypoplasia (P2) and mild white matter alterations (P1).

From a structural modeling perspective, *in silico* calculations suggest distinct biophysical perturbations: Thr150 and Tyr172 are situated near the GTP-binding and intradimer interfaces, where substitutions (T150I and Y172D) are predicted to alter regional stability and potentially interfere with longitudinal protofilament assembly; in contrast, P175L and M377I locate to loop or intermediate domains that may impact lateral lattice contacts. However, because each of these variants was identified in only a single patient (n = 1) and direct functional assays were not performed, these computational predictions must be regarded strictly as mechanistic hypotheses. es. The specific neuroimaging abnormalities observed in individual patients cannot be directly or exclusively assigned as fixed phenotypic consequences of these individual variants, as broader genetic background and developmental timing may also contribute. Further cellular and functional studies are necessary to evaluate whether these specific structural alterations translate into distinct cellular tubulin phenotypes.

TUBB2A is a member of the β-tubulin family, encoding neuron-specific β-tubulin. It is involved in key processes such as microtubule assembly, neuronal migration, axonal transport, and mitotic spindle formation (Sferra et al., 2018). To date, 28 cases of TUBB2A variants have been reported in the literature (Schmidt et al., 2021; Brock et al., 2021). Unlike the traditionally considered static defects in some tubulinopathies, published literature has reported that specific *TUBB2A v*ariants can exhibit “dynamic deterioration” (Cai et al., 2020; Schmidt et al., 2021)—such as the p. Asp417Asn variant causing progressive neurodegeneration resembling complex spastic paraplegia via defective KIF1A-mediated axonal transport (Sferra et al., 2018). In contrast, within our cohort, developmental regression was documented exclusively in two individuals harboring *TUBA1A* variants (P1 and P3), whereas none of the four *TUBB2A* patients exhibited dynamic regression or secondary loss of milestones during their follow-up period. This clinical discrepancy between our cohort and previously reported neurodegenerative *TUBB2A* cases may be attributable to allele-specific pathomechanisms (as our cases carried variants A248V, D203E, R262H, and R391H rather than C-terminal kinesin-binding domain alterations) as well as the relatively young age and shorter duration of follow-up age. Patients carrying the same site also have phenotypic heterogeneity. For instance, the MRI findings of the hotspot variant A248V in *TUBB2A* can range from focal to diffuse pachygyria (Schmidt et al., 2021). In this study, the phenotype of patients carrying this mutation was different from that reported in the literature, with normal MRI findings.

In this study, we describe four additional patients, including three loci (D203E, R262H, R391H) not previously reported in the literature (Schmidt et al., 2021; Brock et al., 2021). As a member of the MTs gene family, craniocerebral MRI findings represent the most crucial phenotypic feature of *TUBB2A* variants. A synthesis of literature data and our own MRI findings across 32 patients revealed that 29 (90.6%) exhibited MRI abnormalities. Core imaging features included lateral ventricle enlargement (16/29, 55.1%), corpus callosum abnormalities (17 cases, 58.62%), and cortical dysplasia (16/29, 55.1%). Additionally, *TUBB2A* can present specific manifestations such as right caudate nucleus hypoplasia and periaqueductal gray matter signal abnormalities (Schmidt et al., 2021; Brock et al., 2021).

As a member of the β-tubulin family, *TUBB4A* can cause neurodevelopmental disorders characterized by leukodystrophy, with significant clinical phenotypic heterogeneity (Ben Jdila et al., 2023; Xiao et al., 2022). In this study, P10 harbored a large *de novo* 2.36 Mb chromosomal 19p13.3–p13.2 duplication (GRCh38/hg38, chr19:4816250–7174,742). Because this duplicated region encompasses several neighboring genes alongside *TUBB4A*, the observed phenotype—consisting of early-onset developmental delay, focal seizures, and cerebral volume changes—cannot be exclusively attributed to *TUBB4A* in isolation. (Ben Jdila et al., 2023). Instead, it represents a contiguous gene duplication syndrome. Future studies dissecting smaller, overlapping microduplications or specific point mutations will be essential to precisely delineate the contribution of *TUBB4A* versus adjacent dosage-sensitive genes within this interval. The two patients in this study (G10S and a chromosome 19 fragment duplication) exhibited distinct onset patterns, and the G10S variant was first reported here. Based on genotypic and motor milestone classification, both cases were classified as late-infantile form, characterized by slowly progressive motor impairment. Notably, both patients presented with focal epilepsy and EEG showing Rolandic area discharges, a feature rarely described in previous *TUBB4A*-related literature (Xiao et al., 2022). Additionally, the patient harboring the G10S variant presented with concurrent systemic lupus erythematosus (SLE). Given that this finding is documented in only a single proband (n = 1), it should be interpreted strictly as an unusual clinical observation and potentially a coincidental event, rather than definitive evidence that immune dysfunction is an integral component of the *TUBB4A* phenotypic spectrum. SLE is a complex autoimmune disorder with multifactorial and polygenic susceptibility, particularly in young female individuals; therefore, an incidental co-occurrence cannot be excluded. Although the microtubule cytoskeleton is involved in fundamental leukocyte dynamics and immunological synapse assembly, any putative role of *TUBB4A* variants in predisposing to autoimmune dysregulation remains entirely speculative. Further systematic screenings in larger patient cohorts and dedicated immunobiological assays will be necessary to clarify whether this co-occurrence is purely coincidental or reflects an extremely rare manifestation (Chen and Yan, 2022).

TUBGCP6 encodes a core subunit of the γ-tubulin ring complex (γ-TuRC), which orchestrates microtubule nucleation and spindle pole integrity. Biallelic pathogenic loss-of-function variants in *TUBGCP6* are characteristically associated with autosomal recessive microcephaly with chorioretinopathy 1 (MCCRP1) (Thomas-Wilson et al., 2023). In proband P11, trio-WES uncovered a maternal missense alteration (c.5050A>G, p. Ile1684Val) paired with a paternal non-canonical intronic alteration (c.1492-35G>A). While *in silico* algorithms such as SpliceAI predicted a negligible splicing impact, non-canonical splice and deep-intronic variants that receive conservative or benign automated *in silico* ratings may nevertheless exert subtle, partial effects on transcript processing, pre-mRNA maturation, or translational efficiency. Such alterations can behave as hypomorphic alleles. In this context, the combination of a destabilizing missense allele (p.Ile1684Val, FoldX ΔΔG 0.975 kcal/mol with a leaky hypomorphic intronic allele could maintain a residual level of functional TUBGCP6 protein expression. This dosage-sparing mechanism offers a rational biological explanation for the milder clinical manifestation observed in P11—characterized by microcephaly, short stature, and speech delay, without chorioretinopathy, seizure episodes, or structural brain dysplasia typical of classical biallelic null states. Nonetheless, we acknowledge the absence of patient-derived RNA sequencing or *in vitro* minigene reporter assays as a limitation in this case. Consequently, the pathogenicity of c.1492-35G>A remains classified as a variant of uncertain significance (VUS), and the compound heterozygous causality in P11 is considered provisional pending experimental transcriptional validation. To further address the potential regulatory impact of the paternal non-canonical intronic variant (c.1492-35G>A, chr22:50226877C>T [GRCh38]), we additionally incorporated predictions from the AlphaGenome framework and the AlphaGenome Variant Impact (AVI) score. Consistent with the very low SpliceAI delta scores, the AlphaGenome predictions yielded a similarly low AVI score, indicating a lack of robust computational evidence for major disruption of canonical splicing or profound non-coding regulatory perturbation. While we previously hypothesized that this allele might act as a leaky hypomorphic variant cooperating with the maternal missense allele (p.Ile1684Val) to confer a milder phenotype, we fully acknowledge that—in the absence of patient-derived RNA sequencing, minigene reporter assays, or quantitative transcript validation—this c effect remains strictly hypothetical. Consequently, the pathogenicity of c.1492-35G>A is appropriately maintained as a variant of VUS, and the compound heterozygous causality in P11 is considered provisional.

Our *in silico* structural analyses provide theoretical models illustrating how missense substitutions in tubulin and associated proteins might perturb local conformation, alter hydrogen bond networks, and shift thermodynamic stability (Maillard et al., 2023). For example, the substantial energy increase calculated for *TUBA1A* p. Tyr172Asp (ΔΔG 5.15 kcal/mol) and the altered bonding predicted for *TUBB2A* p. Arg262His are interpreted as theoretical indicators of potential structural destabilization, rather than direct evidence of functional impairment. It is important to emphasize that while these computational models offer plausible hypotheses regarding impaired GTP binding, dimer interaction, or lattice dynamics, they represent theoretical predictions rather than validated biological mechanisms (Maillard et al., 2023; Hebebrand et al., 2019).

A critical limitation highlighted by the wide range of predicted free energy changes (from approximately 0.50 kcal/mol for *TUBA1A* T150I to 5.15 kcal/mol for Y172D) is that the magnitude of calculated thermodynamic destabilization does not exhibit a direct, linear correlation with clinical severity, epilepsy refractoriness, or the presence of cortical malformations. For instance, variants with relatively high predicted ΔΔG shifts and distinct local bonding alterations manifested with diverse neuroimaging outcomes ranging from normal scans to lissencephaly. Consequently, these structural models must be interpreted strictly as exploratory biophysical hypotheses regarding local protein conformation rather than quantitative predictors of clinical phenotypes. Several methodological limitations inherent to AlphaFold3 and FoldX must be acknowledged when interpreting these *in silico* predictions. First, FoldX utilizes empirical force fields parameterized primarily for soluble globular proteins and static crystal structures, which may not accurately capture the complex thermodynamic landscape of multimeric, highly dynamic cytoskeletal polymers like microtubules. Second, AlphaFold3 generates static structural models based on deep learning trained on public structural databases, but it does not simulate real-time conformational fluctuations, GTP/GDP nucleotide hydrolysis states, or crucial physiological interactions with tubulin-specific co-factors and molecular chaperones in the crowded cellular cytoplasm. Consequently, the calculated ΔΔG values and predicted local hydrogen bond alterations must be viewed as preliminary biophysical estimates rather than definitive quantitative measures of protein destabilization, and they cannot substitute for direct experimental verification.

A clear distinction must be maintained between the observed clinical phenotypes (such as developmental delay, drug-resistant seizures, and neuroimaging findings) and these putative biophysical impacts. Given the sample size constraint where each variant is represented by a single proband, the apparent correlation between residue location and phenotypic severity remains observational. Dedicated functional assays—such as *in vitro* microtubule polymerization kinetics, neuronal migration assays, and tubulin heterodimer incorporation experiments—are indispensable before mechanistic causality can be firmly claimed.

In conclusion, this study analyzed 11 patients harboring variants in *TUBA1A*, *TUBB2A, TUBB4A*, and *TUBGCP6*. Our findings underscore that although global developmental delay is a consistent hallmark (11/11, 100%), epilepsy and structural brain malformations exhibit variable penetrance and substantial phenotypic diversity (present in 81.8% and 63.6% of patients, respectively, with normal brain MRI documented in over one-third of the cohort). At the molecular level, *in silico* modeling suggests that these missense substitutions may potentially affect protein stability and conformation, thereby hypothesized to theoretically perturb microtubule dynamics—a premise that warrants rigorous future experimental validation. Newly identified several mutation sites such as *TUBA1A* P175L and *TUBB4A* G10S expand the genotype-phenotype spectrum. Several limitations of the present study should be noted. First, the cohort size is inherently limited, and crucially, each novel variant was documented in a single proband (n = 1), precluding definitive conclusions regarding strict genotype-phenotype correlations. Consequently, the clinical and neuroimaging presentations described herein reflect observed individual phenotypes rather than universally generalizable variant-specific signatures. Second, given the preliminary nature of our *in silico* approaches, our structural insights are derived solely from computational predictions (AlphaFold3 and FoldX). Although these provide useful hypotheses regarding protein instability, they do not constitute direct biological proof of disrupted microtubule dynamics, axonal transport, or neuronal migration. Larger multicenter international registries and complementary functional validations—including live-cell imaging of microtubule networks, patient-derived induced pluripotent stem cell (iPSC) neuronal models, and *in vivo* animal assays—are warranted to definitively dissect the pathogenic mechanisms and validate variant-specific phenotypic consequences.

## Data Availability

The datasets presented in this study can be found in online repositories. The names of the repository/repositories and accession number(s) can be found in the article/supplementary material.
